# Design and clinical application of injectable hydrogels for musculoskeletal therapy

**DOI:** 10.1002/btm2.10295

**Published:** 2022-03-15

**Authors:** Øystein Øvrebø, Giuseppe Perale, Jonathan P. Wojciechowski, Cécile Echalier, Jonathan R. T. Jeffers, Molly M. Stevens, Håvard J. Haugen, Filippo Rossi

**Affiliations:** ^1^ Department of Chemistry, Materials and Chemical Engineering “Giulio Natta” Politecnico di Milano Milano Italy; ^2^ Department of Biomaterials Institute of Clinical Dentistry, University of Oslo Oslo Norway; ^3^ Material Biomimetic AS Oslo Science Park Oslo Norway; ^4^ Industrie Biomediche Insubri SA Mezzovico‐Vira Switzerland; ^5^ Faculty of Biomedical Sciences University of Southern Switzerland Lugano Switzerland; ^6^ Ludwig Boltzmann Institute for Experimental and Clinical Traumatology Vienna Austria; ^7^ Department of Materials Imperial College London London UK; ^8^ Department of Bioengineering Imperial College London London UK; ^9^ Institute of Biomedical Engineering Imperial College London London UK; ^10^ Hybrid Technology Hub, Centre of Excellence Institute of Basic Medical Science, University of Oslo Oslo Norway; ^11^ Department of Mechanical Engineering Imperial College London London UK

**Keywords:** bone regeneration, cartilage regeneration, clinical translation, hydrogels, medical devices, regenerative medicine, viscosupplementation

## Abstract

Musculoskeletal defects are an enormous healthcare burden and source of pain and disability for individuals. With an aging population, the proportion of individuals living with these medical indications will increase. Simultaneously, there is pressure on healthcare providers to source efficient solutions, which are cheaper and less invasive than conventional technology. This has led to an increased research focus on hydrogels as highly biocompatible biomaterials that can be delivered through minimally invasive procedures. This review will discuss how hydrogels can be designed for clinical translation, particularly in the context of the new European Medical Device Regulation (MDR). We will then do a deep dive into the clinically used hydrogel solutions that have been commercially approved or have undergone clinical trials in Europe or the United States. We will discuss the therapeutic mechanism and limitations of these products. Due to the vast application areas of hydrogels, this work focuses only on treatments of cartilage, bone, and the nucleus pulposus. Lastly, the main steps toward clinical translation of hydrogels as medical devices are outlined. We suggest a framework for how academics can assist small and medium MedTech enterprises conducting the initial clinical investigation and post‐market clinical follow‐up required in the MDR. It is evident that the successful translation of hydrogels is governed by acquiring high‐quality pre‐clinical and clinical data confirming the device mechanism of action and safety.

## 1 HYDROGELS AS MEDICAL DEVICES

Hydrogels represent a group of biomaterials consisting of water‐swollen polymer or colloidal networks.^1^ Hydrogels are viscoelastic materials that have attracted attention in regenerative medicine due to their ability to structurally mimic the extracellular matrix (ECM),^2^ thereby creating a conducive environment for cell proliferation and tissue regeneration. The viscoelastic properties of hydrogels allow them to function as stem cell carriers or scaffolds for controlled drug release. Within the review by Correa and colleagues,^3^ these applications of hydrogels are discussed in the context of clinical translation. From a regulatory perspective, hydrogels can be considered medical devices if their therapeutic effect comes from their intrinsic structure, because their physical, chemical, or mechanical effects are the primary mechanism of action for their therapeutic function. To be classified as medical devices hydrogels cannot have any medicinal component, effect, or mechanism of action. For the case of medical devices, harmonized in the European market, the relevant regulation is EU 2017/745, which entered in force on May 26, 2021. Melvin and Torre^4^ have discussed the rationale behind the new regulation. Recently, Catoira et al.^5^ have discussed how the regulation affects the translation of hydrogels. Hence, this review will consider how hydrogels can be designed to satisfy these regulations. However, expanding cells or integrating cell‐stimulating therapeutics into the medical device results in these hydrogel systems being regulated as medicinal products (drugs/biologics). Indeed, they are considered as drugs when their principal mode of action is pharmacological, metabolic, or immunological.^6^ The consequence of the medicinal regulation is that a more thorough investigation of the biocompatibility and therapeutic effect is required before such solutions can be approved for clinical application. This increases the translational barriers and the time before patients can benefit from the treatment. Therefore, it is attractive to translate hydrogels solutions as medical devices such that the therapy can reach the clinic earlier and is more affordable. A detailed discussion of the classification can be found in Section 4.

Particularly in applications for musculoskeletal disorders, there is an unmet need for minimally invasive therapies, where the use of injectable hydrogels has tremendous potential. The demand is driven by an aging population that gives two unique opportunities: (1) an increasing number of patients outlive the longevity of permanent medical devices; thus, hydrogel therapies can be used to delay permanent implantation, (2) minimally invasive therapies give a treatment opportunity for the growing population group that would otherwise not survive the trauma induced by conventional surgeries.^7^ Examples of these type of devices are represented by hydrogels for joint lubrication,^8^, ^9^ injectable scaffolds for guided bone^10^ or cartilage regeneration,^11^ or nucleus pulposus (NP) replacements.^12^ These are widely different applications for diverse tissues with different loading modes and levels. Consequently, a one‐fit‐all hydrogel is an unlikely strategy, and thought should be put into the clinical requirements of the material when designing the hydrogel.

## 2 HYDROGEL DESIGN

**FIGURE 1:**
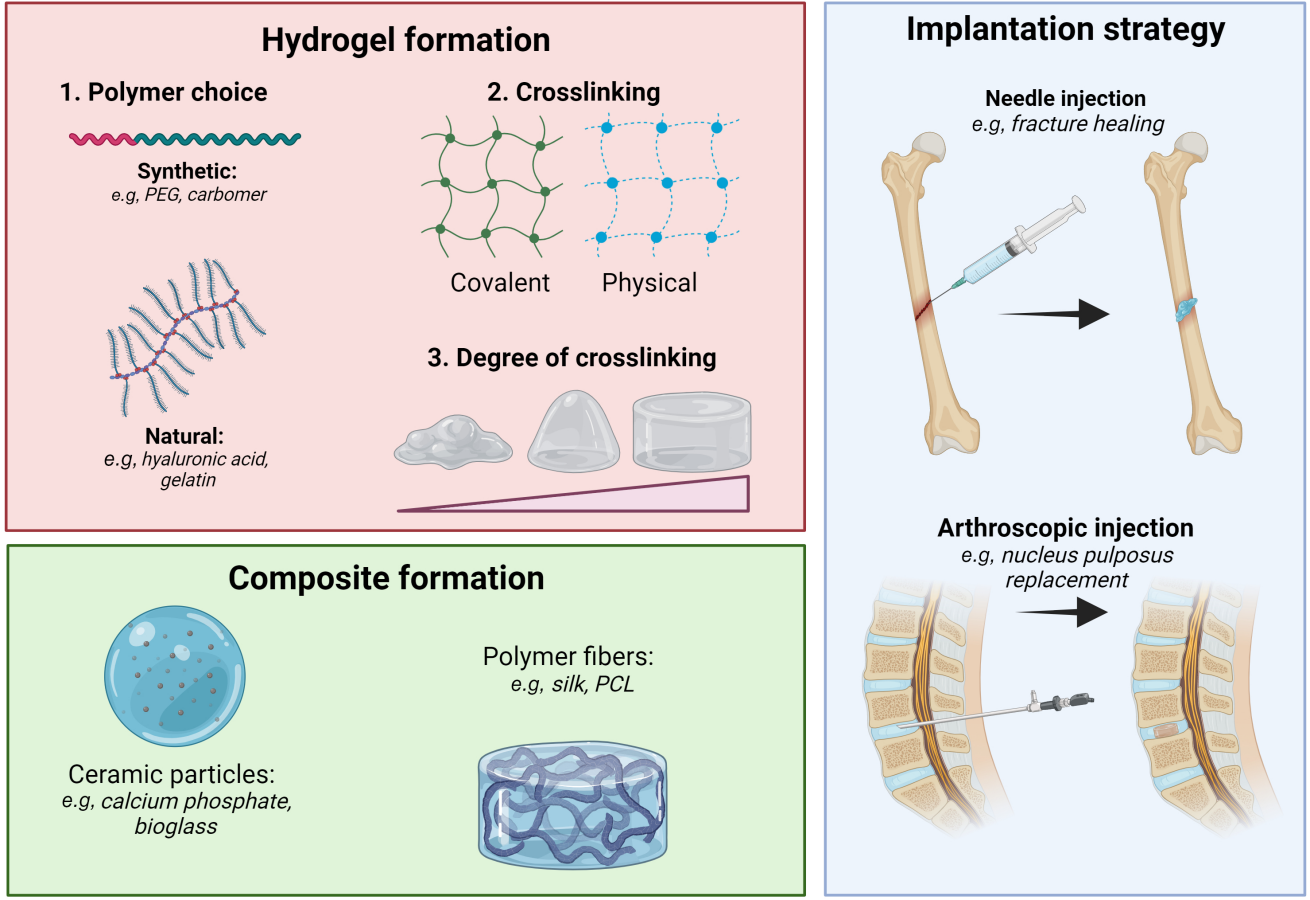
Schematic of the design process of hydrogels as medical devices for musculoskeletal application. The process consists of three design blocks, first, the hydrogel is developed, then any particles or other composite inclusions are added before the delivery strategy is chosen

Hydrogels are an extensively investigated class of biomaterials, and an increasing number of products have reached the clinic. In the following section, we will go through the design steps of the hydrogels and discuss what considerations need to be taken to improve the likelihood of clinical translation and to comply with the European Medical Device Regulations (MDRs). The design process is summarized in Figure 1.

### 2.1 Material selection

The first step in the design process is to select a suitable polymer to form the hydrogel. There is a larger group of naturally derived polymers such as collagen,^13^ hyaluronic acid (HA),^14^ chitosan,^15^ cellulose,^16^ and alginate.^17^ Although not exclusively, plant‐based polymers tend to be composed of saccharides, such as cellulose, and animal‐based polymers tend to compose of protein, for example, collagen.^18^ These have been attractive as their natural origin makes them favorably biocompatible and biodegradable but can introduce issues such as immunogenicity and limited mechanical properties.^19^ From a translational perspective, these are limited by high cost and batch‐to‐batch variability.^5^, ^19^, ^20^ Alternatively, synthetic polymers such as poly(ethylene glycol) (PEG),^21^ poly(vinyl alcohol),^22^ poly(acrylic acid),^23^ and poly(acrylamide)^24^ can be used. Synthetic polymers are industrially more used as they are more favorable from both cost and regulatory perspective, the two being also connected. Synthetic polymers can be produced in more robustly repeatable manners and more efficiently with respect to naturally derived ones, making them readily scalable.^25^ Synthesis is typically a more straightforward production process and ensures controlled environmental factors thus limiting the risk of contamination. Synthetic polymers are favorable versus naturally derived raw materials as they allow for improved traceability and higher degree of availability which finally reduces the cost.^25^, ^26^ However, their clinical adoption has been limited, and those that exist usually provide a mechanical mechanism of action, for example, PEG‐hydrogel as a spacer between prostate and rectum to protect the rectum during radiotherapy.^27^ For the regenerative market, the translation is insignificant, which has been attributed to their low biocompatibility.^28^ The low biocompatibility is likely related to lack of cell‐specific bioactivity, including cell adhesive and migratory cues, and cell‐mediated material degradation.^29^ This highlights how biocompatibility is vital for the success of any hydrogel, and the biological response should be central to the choice of the polymer for the hydrogel. Implanted materials can either integrate physiologically, leading to minimal or no scaring, or the material can induce chronic inflammation and a foreign body response.^30^ After injection, the material must provide appropriate biochemical and biophysical signals to recruit host cells that will eventually produce new native tissue.^31^ Immune cells also play a key role in the signaling cascade leading to tissue regeneration, and appropriate engineering of the local immune response can boost the tissue regeneration.^32^ For instance, monocytes and macrophages releasing cytokines including BMP‐2, BP‐4, and TGF‐β1 support osteoblast differentiation and proliferation.^33^ The current gold standard for understanding biocompatibility remains clinical trials, but essential information can also be derived from well‐designed pre‐clinical trials. When selecting the polymer, we have two conflicting interests; from a biological perspective, natural biopolymers are favorable due to higher biocompatibility, meanwhile synthetic polymers have more controllable properties, including swelling, degradation, phase transitions, and mechanical properties.^34^ Additionally, synthetic polymers are more favorable from a regulatory and financial perspective. To balance these interests co‐gel solutions such as PEG‐HA^35^ and gelatin methacrylate‐PEG diacrylate^36^ are promising strategies at combining features from both groups of polymers. Moreover, synthetic polymers can be functionalized with proteins and peptides to improve cell attachment and proliferation. For instance, the inclusion of RGD‐peptides in PEG gels has demonstrated these capabilities on multiple cell types.^37^, ^38^

### 2.2 Crosslinking/gelation

The next step is to form the gel‐network by crosslinking the polymer chains. There are two options here, and the polymer can be physically or chemically crosslinked. Physical crosslinking is a reversible process where weak non‐covalent interactions (e.g., van der Waals, hydrogen bonding, electrostatic interactions) keep the network stable. The advantage here is that the gel can be formed without using a crosslinking agent and the gel is easier to mold into the defect geometry.^39^ Alternatively, chemical crosslinking (covalent bonds) can be used. The covalent bonds tend to convey to the gel's improved mechanical properties and higher stability.^40^ The gel stability is a vital matrix design as long degradation times and the inability to be remodeled by the cells will hamper tissue growth. In contrast, a fast degradation time will leave an unfilled void after the gel degradation.^41^ The degree of crosslinking, meaning the number of bonds that interconnect the polymers to each other, is an important parameter for the material properties. With a higher degree of crosslinking, we can expect a higher viscosity, stiffness, and longer degradation time.^42^, ^43^ It has early been established that with increased degree of crosslinking, the gel's ability to swell decreases.^44^ The equilibrium degree of swelling affects a series of properties such as solute diffusion coefficient, mechanical properties, and the mobility of therapeutic agents.^45^ From a translational perspective, chemical crosslinking means introducing new chemicals and at least one more chemical reaction. It must be proven that the biomaterial remains biocompatible and that there is not an increase of leachables such as unreacted crosslinker. Dialysis tends to be an efficient method for removing such impurities, but the removal and biocompatibility must be proven. This is further discussed in Section 5.

### 2.3 Composite design

Like with other biomaterial types, composite materials can be formed using hydrogels. This is a favorable strategy as the final material will have inherent properties from base materials in addition to the properties derived from the interaction between material components. In terms of hydrogels, this could be the introduction of fibers, for instance, to improve mechanical properties or guide tissue growth, or particles, for example, a ceramic phase which can boost bone regeneration. Li and co‐workers^46^ combined a hydrogel of thiolated HA and polyethylene glycol diacrylate (PEGDA) gel covalently crosslinked to fragmented, electrospun polycaprolactone fibers. The fibers gave improved mechanical properties compared to the HA‐PEG gel alone, thereby they could mimic the mechanical properties of native fat tissue. Moreover, their in vivo trials with subcutaneous injection of the material in a rat and a rabbit model suggest improved macrophage polarization toward a pro‐regenerative phenotype and enhanced angiogenesis.

The inclusion of an inorganic phase in the polymeric hydrogel material has been a popular strategy for bone regeneration. Chahal and colleagues^47^ developed a PEG hydrogel with amorphous calcium phosphate particles. They demonstrated that the particles both gave a higher stiffness, and slowly released calcium and zinc ions into the solution, creating conducive properties for bone regeneration. Although they observed a qualitative increase in gel mineralization, they could not demonstrate statistical significance. Furthermore, the human mesenchymal stem cells (MSCs) they used were unable to attach to the gel before they functionalized the PEG with RGD tripeptide motifs. This highlights the importance of choosing a polymer with high bioactivity to succeed clinically and demonstrates one of the shortfalls of most fully synthetic systems. Semi‐synthetic systems, however, are promising as they allow for tunable properties such as gelation mechanism and adhesion to tissues. Researchers from the Langer lab^48^ developed a cellulose hydrogel with PEG‐block‐poly(lactic acid) nanoparticles as non‐covalent crosslinking nodes that gave the gel shear‐thinning and self‐healing properties. In vivo in mice (subcutaneously in the back) they demonstrated biocompatibility with a mild neutrophil‐induced inflammation at day 3 and clearance by macrophages from day 7. A consistent release pattern was observed when particles were loaded with model dual‐hydrophobic/hydrophilic drugs. Wang and colleagues^49^ formed a mechanically strong, transparent, and self‐healing hydrogel by coating clay nanosheets with sodium polyacrylate and physically crosslinking it with dendritic G2 binder.

Any inclusion will make it, from a regulatory perspective, a completely new biomaterial. Therefore, it will require the standard omni‐comprehensive testing due for any novel formulation. This includes the application of ISO 10993 family of standards that encompass biocompatibility testing up to clinical studies, and for biodegradable biomaterials the documentation requirements that degradation products do not accumulate in any body organs.^50^

### 2.4 Implantation method

Although the implantation method of the hydrogel is not directly a design variable affecting the gel properties, the hydrogel should be designed with implantation feasibility in mind. There are primarily two strategies of implantation in current use. The traditional is surgical incision implantation, where a surgeon cuts a flap through the patient's dermis and physically places the implant at the desired location. The advantage of this intervention is that the gel can be pre‐shaped prior to the surgery and have higher mechanical stiffness. The disadvantage is that the incision surgery gives a longer hospitalization time, longer recovery time, increased postoperative pain,^51^ and higher risk of bacterial infections.^52^ Therefore, injectable solutions are attractive minimally invasive strategies that give less trauma,^53^ less blood loss, shorter surgeries, and rapid recovery.^54^ This brings its own technical challenges, as the gel must have low enough viscosity to be injectable through a needle or arthroscopic instruments. To have adequate viscosity during injection, it might be favorable to use a low degree of crosslinking,^43^ a physically crosslinked gel exhibiting shear‐thinning properties,^48^, ^55^ or utilize in situ crosslinking of the hydrogel using methods such as click‐chemistry,^56^ ultrasound,^57^ and photoinitiated crosslinking.^11^, ^12^ For in situ crosslinking, it is imperative to ensure that there are no adverse chemical reactions between the material and the surrounding biological tissue. For instance, thiol groups are naturally occurring in the body, so if a thiol‐based Michael addition strategy is used for gelation, there is a risk of undesired cross‐reactivity, oxidation, or metabolism.^40^ This has inspired the focus on bioorthogonal chemistry, a class of high‐yielding reactions based on selective transformation not commonly found in biology.^58^

An innovative solution for injection of a hydrogel therapy is the Flowbone® solution developed by researchers at EPFL in Switzerland. They have developed a biphasic gel solution for bone regeneration where the first phase consists of covalently crosslinked HA with hydroxyapatite particles incorporated, that is carried in a second aqueous phase comprising more hydroxyapatite particles.^59^ The biphasic system allows a low viscosity and thereby injectability. This solution also allows for the loading drugs such as bisphosphonates,^60^ which is now under investigation in pre‐clinical trials.

Other solutions chose a tactic where the crosslinking occurs in situ, such as Regentis Biomaterial's GelrinC®, which is discussed below. The in situ strategy allows for a low viscosity during injection, while the high viscosity and mechanical properties are obtained after injection.

## 3 APPLICATIONS

A series of hydrogel‐based products have been approved for clinical use in the EU and the United States, particularly for viscosupplementation (VS) in joints for osteoarthritis (OA). Furthermore, regenerative gels are now emerging that in addition to providing temporary pain relief and functional improvement, attempt to regrow or support the regrowth of the tissue for a longer‐lasting therapeutic effect. In this section, we describe some of the leading clinical products for VS. In addition, we will discuss the products that have undergone clinical trials or been commercialized in the EU or the United States to regenerate bone, cartilage, or NP tissue. The products we will discuss are summarized in Table 1. We present their application indications, therapeutic effect, delivery method, and composition. Apart from VS, the list is exhaustive to the authors' best knowledge but might suffer from lack of data availability as many manufacturers choose to keep data on file rather than publishing their results. With the introduction of the European EUDAMED database, this is expected to change within the EU market. Bone putties (DBM/inorganic particles in hydrogel carrier) have been excluded for bone regeneration products unless they are marketed as injectable gels.

**TABLE 1:** Clinically available injectable hydrogel products for treatments of musculoskeletal indications

Indication and treatment mode	Product (manufacturer)	Composition	Delivery method	Therapeutic claim	CE/FDA approvals
Osteoarthritis					
VS	Gel‐One®^61^, ^62^ (Zimmer Biomet)	Cinnamic acid functionalized HA crosslinked with UV light	Single 3 mL injection	Pain relief up to 26 weeks	FDA
	Orthovisc®^63^ (Anika Therapeutics)	SHA (1.0–2.9 MDa) in saline water	Three separate injections of 30 mg in 2 mL solution	Pain relief up to 6 months	FDA
	Monovisc®^64^ (Anika Therapeutics)	High MW HA, lightly crosslinked with biscarbodiimide	One 4 mL injection	6 months pain relief	FDA
	Hymovis®^65^ (Fidia Farmaceutici)	HA 500–730 kDa, functionalized with 2–3% hexadecylamine	Two times 3 mL at a week interval	Lubrication and pain relief up to 12 months	FDA
	Arthrosamid®^66^, ^67^ (Contura International)	2.5% polyacrylamide in sterile water—non‐degradable	Single session injection of 6 × 1 mL through 21G cannula (syringes replaced using luer‐lock system)	Long‐lasting pain relief (52 weeks proven)^a^	CE & Clinical Trials (US)
Regeneration	BST‐CarGel®^68^, ^69^ (Smith & Nephew)	Chitosan dissolved in saline water and autologous blood	Mini‐arthrotomy or arthroscopy in combination with bone marrow stimulations such as microfracture	Superior hyaline cartilage regeneration compared to microfracture alone	CE
	RegenoGel®^70^ (ProCore)	HA (1.6 MDa) conjugated to purified platelet‐rich plasma‐derived fibrinogen	Synovial fluid is first removed through a 21G needle, before 4 mL of gel is injected. Two administrations 3 months apart	Pain relief and cartilage regeneration	Clinical trials (EU and US)
	GelrinC®^71^, ^72^, ^73^ (Regentis Biomat.)	PEGDA with denatured fibrinogen	Injected after microfracture, crosslinked using UVA light	Degrades over 6–12 months while being replaced by regenerated cartilage	Clinical trials (EU and US)
Bone defects^b^	DDD				
Regeneration	Emdogain®^74^, ^75^ (Straumann)	Porcine EMD in propylene glycol alginate gel [30 mg/m]	Flap incision or flapless injection in dental application	Regenerates periodontal tissue (cementum, periodontal ligament, bone)	CE & FDA
	Perioglas®^76^ (NovaBone)	Calcium phosphosilicate particles + a PEG and glycerine gel‐like binder	Either as a moldable putty or through syringe injection	Dental bone regeneration	CE & FDA
	Actifuse®^77^, ^78^ (Baxter)	Phase‐pure silicon‐substituted calcium phosphate in poloxamer 407	Injectable through syringe	Bone void filler in spinal and orthopedic application	CE & FDA
	Dynagraft III®^79^ (Integra)	DBM in poloxamer carrier	Injectable through syringe or delivered as a putty	Bone void filler	FDA
	AlphaGRAFT® (Alphatech)	DBM in poloxamer carrier	Extruded through syringe	Bone void filler	FDA
	AlloFuse®^79^ (AlloSource)	29% allographic DBM in polyethylene oxide polypropylene oxide block copolymer	Mixed with autologous bone for spinal fusion or injected in trauma cases	Void filler, graft extender	FDA
	Optium®^79^ (LifeNet Health)	Allographic DBM in glycerol	Allographic DBM in glycerol	Bone graft extender and void filler	FDA
	Grafton DBM® gel^80^, ^81^ (Medtronic)	Allographic DBM in glycerol	Mixed with autologous bone for spinal fusion or injected in trauma cases	Bone graft extender and void filler	FDA
	Tactoset®^82^ (Anika Therapeutics)	HA carrier with calcium phosphate	The HA and CaP are mixed then injected. Hardens within 15–20 min	Bone void filler for orthopedic application	FDA
	Kinex® Bioactive Gel^83^ (Globus Medical)	Bioglass, collagen and HA	Injectable solution	Bone void filler	FDA
DDD					
Nucleus pulposus replacement	GelStix®^84^ (Replication Med.)	Polyacrylonitrile	Injected through a 22G needle and swells in situ	Pain relief from 1 month after surgery for at least 12 months	CE
	HYADD4‐G®^85^, ^86^ (Fidia Farmaceutici)	HA 500–730 kDa, functionalized with 2%–3% hexadecylamine	1.5 mL [8 mg/mL] intradiscal injections guided by X‐ray	Statistically significant pain relief up to 24 weeks	Clinical Trials
	BioDisc®^87^, ^88^ (CryoLife)	Albumin + glutaraldehyde hydrogel	Crosslink in situ within 2 min	Reduction in pain after 6 months	Unknown^c^
	NuCore®^89^, ^90^ (Spine Wave)	Block polymers of silk and elastin crosslinked in situ with diisocyanate	Injected with a sealed vented needle to recover disc height (0.3–1.9 mL)	Reduction in back and leg pain, regained disc height	Unknown^c^

*Note*: The list of products for viscosupplementation (VS) is non‐exhaustive due to many products on the market. Instead, representative products for different materials and crosslinking mechanisms are presented. The other types of products are exhaustive to the authors' best knowledge.

Abbreviations: CaP, calcium phosphate; DBM, demineralized bone matrix; DDD, degenerative disc disease; EMD, enamel matrix derivatives; FDA, Food and Drug Administration; HA, hyaluronic acid; PBS, phosphate‐buffered saline; SHA, sodium hyaluronate; VS, viscosupplementation.

^a^ Clinical trial has a 5‐year follow‐up period.

^b^ Bone defects = trauma, oncology, craniofacial.

^c^ Seems to be discontinued.

Many manufacturers have chosen to not publish their findings but keep their data privately on file. This applies to the products AphaGRAFT®, Kinex®, AlloFuse®, and Tactoset®, meaning we have limited information on these products which can limit our discussion of these solutions.

### 3.1 Cartilage treatment

An exciting area where injectable hydrogels have become an established treatment is cartilage degeneration in joints. This is primarily indicated by OA, a disease‐causing degeneration of the cartilage and the subchondral bone in the joints and affects roughly a third of people above 65 years,^91^ thereby having a high socioeconomic cost. In addition to degenerated cartilage and subchondral bone, synovitis and systemic inflammation are part of the pathogenesis.^92^ Patients with mild to moderate OA usually are treated with intra‐articular injection of corticosteroids, as it provides an anti‐inflammatory effect.^93^ However, corticosteroids are just capable of treating the symptoms, that is, reducing pain, but not able to stop the progress of OA.^94^ Therefore, VS has become a popular treatment alternative as it provides a longer therapeutic effect.^95^

**FIGURE 2:**
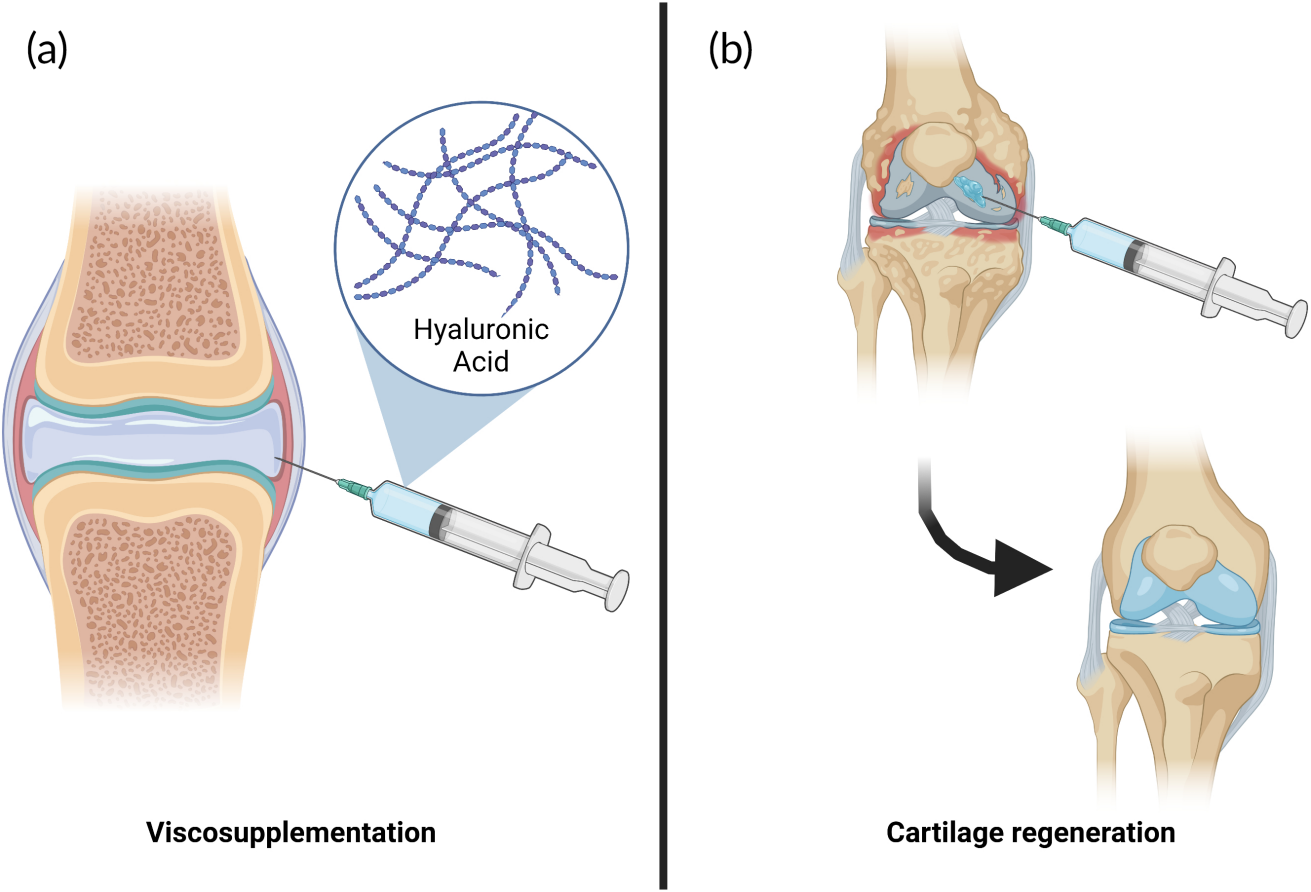
Treatment of cartilage defects caused by osteoarthritis. (a) Viscosupplementation using hyaluronic acid to obtain improved joint movement and pain relief. (b) Cartilage regeneration using injectable hydrogels

For late‐stage OA, arthroplasty is the preferred treatment, where the joint is partially or totally replaced with a prosthesis that is typically made of cobalt chrome or titanium alloys.^96^ An alternative treatment is microfractures to release chondroprogenitor cells to the diseased location, but this tends to form fibrocartilage instead of desired hyaline cartilage.^97^ The fibrocartilage has inferior mechanical properties than the native hyaline cartilage,^98^ providing a temporary solution. Injectable hydrogels have become an attractive strategy for treatment in OA, both for delaying arthroplasty and attempting to regenerate the damaged cartilage toward more native‐like cartilage than what can be achieved from microfracture. The two primary therapies, VS and regeneration, are illustrated in Figure 2.

#### 3.1.1 Viscosupplementation

There are multiple solutions based on HA injection into the knee for pain relief through VS (Table 1). There are two generations of VS products. The first generation consisted of HA solutions dissolved in an aqueous solution. The second generation consisted of crosslinked HA. To maintain injectability for the crosslinked gels, some of these are granulated HA gels chunks (typically less than 80 μm) that are mixed in an aqueous solution. This is for instance the case for Anika's Monovisc®, as can be deduced from its patent.^99^ The clinical efficacy of VS therapies is debated. In a more extensive meta‐analysis including 89 trials with 12,677 patients involved, they could not observe any clinically relevant benefit.^100^ There were, however, indications that high molecular (>6000 kDa) or covalently crosslinked HA could provide a beneficial therapeutic effect.^100^ In contrast, in another meta‐analysis considering only FDA‐approved VS in randomized, saline‐controlled trials (29 studies; 4866 patients; active: 2673, control: 2193) they concluded that these products are safe and effective through 26 weeks in patients with symptomatic OA.^101^ Simultaneously, a consensus of eight European experts on OA discussed the clinical effect of HA in VS: they unanimously agreed that VS is an efficient strategy for managing mild to moderate knee OA, is a cost‐efficient treatment in knee OA, but is not an alternative to surgery in advanced hip OA.^102^

Although most of the solutions are based on HA, it is essential to consider the chemical composition and design of the gel. As mentioned before, high molecular weights and covalent crosslinking seem to be preferable. A higher molecular weight HA is believed to have improved residence time and adhesion to the cartilage providing more lasting lubrication under loading,^103^ while a crosslinked HA gel would degrade slower than a non‐crosslinked HA solution,^104^ giving a longer therapeutic effect. For instance, for a lightly crosslinked VS such as Monovisc®, one injection provides 6 months of therapeutic effect,^64^ compared to three injections for a conventional non‐crosslinked VS such as Orthovisc®. This gives significant indirect cost savings in the form of fewer hospitalization visits and reduced pain to the patient. More importantly, it can reduce the occurrence of more serious adverse events such as pseudoseptic reactions (inflammation and swelling of joint without infection, occurs in 1%–3% of patients) that typically occurs after second or third injection.^105^

Recent clinical trials demonstrated that injection of HA has anti‐inflammatory and antioxidative properties, which can decrease the progression of OA.^106^ This effect seems to be mediated through receptor signaling via binding with cluster determinant 44, toll‐like receptors 2 and 4, intercellular adhesion molecule I, and layilin, providing a multifactorial mechanism.^107^ Additionally, there are indications that high molecular weight HA promotes an anti‐inflammatory response, meanwhile, low molecular weight HA favors an inflammatory response.^107^ Altogether, intra‐articular injections of HA‐based VS have demonstrated an effect, and there is still room to tune the hydrogel composition to obtain solutions providing better lubrication with enhanced therapeutic benefit.

A recent commercialization is VS made from polyacrylamide such as Contura's Arthrosemid®. Arthrosemid® is a gel consisting of covalently crosslinked polyacrylamide, which is non‐degradable.^66^ It was used initially for veterinary application in horses with OA,^108^ but recently the therapeutic effect has been demonstrated to be functional up to 52 weeks in humans.^67^ As the material is non‐degradable, the therapeutic effect is expected to be significantly longer. An in vivo subcutaneous rat model comparing the acrylamide gel to a HA gel as soft tissue fillers suggested significantly different in vivo behavior. The acrylamide underwent cell infiltration by macrophages and fibroblasts and tissue integration, meanwhile, cell infiltration did not occur in the HA gel which was encapsulated by a thin fibrous layer.^109^ The relevance of the model is limited as the study was conducted in a small animal with a subcutaneous application instead of intra‐articular. However, the results may suggest that the clinical mechanisms of HA and acrylamide gels are different.

#### 3.1.2 Cartilage regeneration

Although VS, such as Monovisc® and Orthovisc®, can typically provide pain relief for up to 6 months, they do not regenerate functional cartilage. This has led to an enormous focus on cartilage regeneration, and there is a series of products in clinical trials. They use different tactics for regeneration; conventionally, a microfracture procedure where bone marrow‐derived MSCs are released into the defect site has been used for cartilage regeneration, but with considerable variability and inconsistency.^110^ Both the BST‐CarGel® solution and the GelrinC® build on this procedure by providing the released MSCs with a scaffold for guided cartilage regeneration. Their mechanism differs slightly. The BST‐CarGel® consists of chitosan dissolved in aqueous glycerol phosphate (buffer at physiological pH), that when mixed with blood forms a clot with increased mechanical properties and longer stability.^68^ The capability of chitosan as a hemostatic agent is derived from its poly(cationic) nature that allows it to bind with the negatively charged thrombocytes and erythrocytes in the blood.^111^ A 5‐year follow‐up study for treatment of OA in the knee demonstrated significantly better cartilage regeneration with BST‐CarGel® compared to microfracture alone,^69^ and their animal trials suggest that the gel also regenerates cartilage with increased hyaline characteristics.^112^ GelrinC® on the other hand, is based on PEGDA mixed with denatured human fibrinogen and can be injected in liquid form but solidifies into a gel upon 90 s of UVA irradiation.^72^ It can be used for both chondral and osteochondral lesions and showed statistical improvement compared to the absence of treatment after 24‐months follow‐up.^71^ Their MRI data suggested a zonal variation in the cartilage, which they interpret as the cartilage might be hyaline‐like rather than fibrous.

Although some indications, neither of the solutions has proven to produce native‐like hyaline cartilage in humans. Part of the reason they cannot prove it is that one cannot take histology samples from living patients. Instead, they must use methods such as magnetic resonance imaging (MRI). Unfortunately, clinical MRIs tend to have a moderate resolution, limiting some of the quality of the data used in the analysis.

Although GelinC® and BST‐CarGel® have shown short‐term improvement, the success is governed by the long‐term results, economic viability, and clear improvement from microfracture alone. Frappier and colleagues^113^ demonstrated this by evaluating the economic value of BST‐CarGel® solution versus microfracture alone using Germany as a reference market. Their results suggest that a positive investment return is reached after 4 years and more than €6400 of cost saved over a 20‐year period. Some essential limitations to this study are a lack of long‐term clinical data for BST‐CarGel® versus microfracture, and it only considers cost and not the quality of life of the patient. Nevertheless, the data suggest that it is clinically feasible to use these different solutions along with microfracture. This should motivate other research to develop new solutions with improved efficacy and at lower costs. A key challenge the field should address is successfully regenerating native‐like hyaline cartilage and developing non‐invasive methods that can aid in its characterization in vivo. Most likely some type of agents, such as microfibers or a biomolecule, is required to guide the direction of the tissue regrowth. Furthermore, regrowth should preferably follow the zonal tissue architecture that can be observed in the native articulate cartilage.^114^, ^115^ Ideally, the cartilage should recruit chondrocytes or MSCs without the need for autologous chondrocyte transplantation or microfracture, but there are currently no such solutions to the authors' best knowledge. At the time of this review, microfracture procedures are estimated to cost €4329 and autologous chondrocyte implantation €14,238.^116^ On top of this comes the cost of the hydrogel used. Hydrogel scaffolds that can induce regeneration using only locally recruited chondrocytes can provide considerable cost savings through reduced surgery times and trauma to the patients.

Another trend that starts to arise is VS‐like products with additional regenerative capabilities. An example of this is ProCore's RegenoGel® solution that was commercially approved in Israel in 2016 and recently completed their FDA phase 4 clinical trials. RegenoGel® is based on HA that is mixed with purified platelet‐rich plasma‐derived fibrinogen that conjugates to form an injectable gel.^70^ In their clinical trials, they have been able to demonstrate that the gel is efficient at treating the symptoms of OA, that is, pain and knee stiffness, for at least 6 months after treatment start,^70^ but more detailed studies are required to investigate the long‐term effect and the ability to regenerate cartilage. Nevertheless, their in vivo cartilage‐bone explant mouse model suggests that the material recruits endogenous cells and differentiates them toward a chondrocyte lineage, yielding significant deposition of GAG‐proteins and collagen type 2.^117^ Although promising for cartilage regeneration, they have yet to demonstrate cartilage regeneration in humans.

### 3.2 Bone regeneration

Healthy bone is vital for structural stability in the musculoskeletal system, and defects result in pain, disability, and reduced mobility in individuals. Additionally, the treatment of bone defects is a tremendous burden to healthcare providers, estimating an annual cost of $5 billion in the United States alone.^118^ Even though bone defects are rarely directly mortal, the trauma‐induced can be hard to recover from. If we consider the case of hip fractures, for elder women (>65 years) there is a 10% likelihood of mortality within 3 months of a hip fracture.^119^ Similarly, a larger meta‐analysis demonstrated that the risk of mortality is increased by a 6‐ and 8‐fold the first 3 months after hip fracture for older women and men (>50 years), respectively.^120^ Nor are there any good treatment alternatives in these cases. In fact, another meta‐analysis demonstrated that the mortality rate 1 year after hip fracture surgery is 24.5%,^121^ suggesting two scenarios: (1) the current medical devices do not have an appropriate therapeutic effect for the elderly population, or (2) the current surgical procedure's invasiveness leads to a challenging recovery for elderly patients .

**FIGURE 3:**
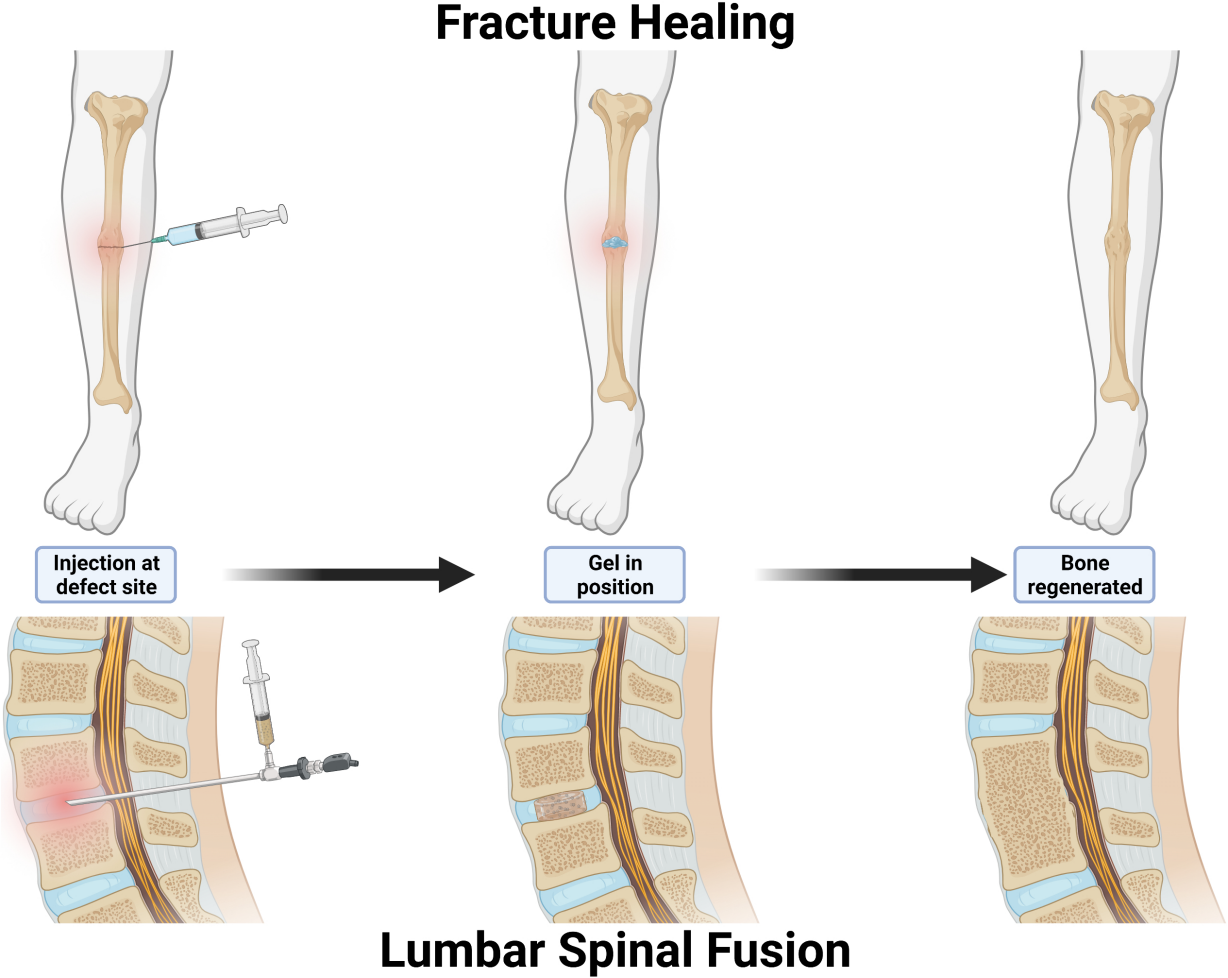
Illustration of hydrogel application for bone regeneration. Top panel: fracture healing in traumatology using a needle‐injected hydrogel. Bottom panel: spinal fusion using arthroscopic injection of a ceramic particle loaded hydrogel

Bone defects can be widely different, and the products used depend on defect size and loading level.^122^ Therefore, this section has been split into three subsections: (1) dental and maxillofacial, (2) trauma and oncology, and (3) spinal fusion. We treat spinal fusion as a separate application as it is the largest application area of bone grafts measured according to market value^123^ and compared with the dental and traumatic and oncologic applications, this is a form of heterotopic ossification. Hydrogels for bone regeneration has been illustrated for two indications in Figure 3.

#### 3.2.1 Dental and maxillofacial

Straumann's Emdogain® dominates the dental market and has more than 20 years of clinical documentation.^124^ Emdogain® is based on a porcine enamel matrix derivative, a cocktail of proteins consisting of amelogenin (90%) and a few other nanomelogenin such as ameloblastin, enamelin, and tuftelin, carried in an aqueous gel solution composed of propylene glycol alginate.^39^ Several of these proteins are identified as intrinsically disordered polypeptides with a one‐to‐many signaling effects in vivo and allow for the formation of multiple tissues in the injection location.^125^ Emdogain® has been proven to regenerate multiple periodontal tissues, including the osseo‐like tissues, acellular cementum,^126^ and alveolar bone,^127^ in addition to connective tissues such as periodontal ligament.^128^ The details of the therapeutic effect of Emdogain® have been discussed in detail in our former review.^39^ A limitation worth noting with Emdogain® is that since it is physically crosslinked, the degradation occurs quicker than it would with a covalently crosslinked hydrogel. The consequence of this is that the mechanical properties degrade quickly, and it can no longer keep the soft tissue flap up, causing a collapse of the gel and limiting the space available for bone regeneration.^129^

Another product that is well established in the dental domain is NovaBone's Perioglas® putty. Initially, it was commercialized as a moldable putty, but a syringe and a cartridge injection system have since been developed. The gel‐like putty consists of calcium sodium phosphosilicate, more specifically Bioglass® 45S5 particles of 32–710 μm diameter, delivered through a gel‐like binder of PEG.^76^ The binder is water‐soluble and is resorbed within 48–72 h after implantation,^76^ hence it is the Bioglass® that has the main therapeutic effect. According to Jones,^130^ the Bioglass® draws its bioactivity from two mechanisms: (1) the accumulation of glass dissolution products provides nucleation sites for a hydroxycarbonate apatite layer that bonds to the surrounding bone. This layer also allows the protein to attach and cells to attach, proliferate and produce ECM; (2) the release of dissolution products also plays an active role in driving osteogenesis through guiding osteoprogenitor cells down an osteoblastic differentiation path, and the osteoblasts are transitioned from a resting stage (G0) to a growth stage (G1). There are, however, concerns regarding inflammatory foreign body reaction around the bioglass particles that might limit the clinical success of the putty.^131^

#### 3.2.2 Orthopedics: Trauma and oncology

An approved product for orthopedics is the Baxter Actifuse® Flow. It consists of silicon substituted calcium phosphate particles of size 90–500 μm carried in an aqueous polymer carrier consisting of poloxamer 407 (P407).^77^, ^78^ The P407 is a triblock polymer with a hydrophobic polypropylene glycol core and hydrophilic PEG side arms, that goes through a thermoreversible gelation mechanism, meaning that the solution gels above a given temperature.^132^ The temperature for which the sol–gel transition occurs decreases with the P407 concentration, and it has been demonstrated that for a concentration of 16.5% (wt.% in purified water) the solution gels at a temperature of 27.1°C.^133^ It can be speculated that the Actifuse® Flow carrier has a P407 concentration of 16.5 wt.% or lower, meaning that it will be liquid at room temperature while at physiological temperatures it would form a gel. The solution has successfully treated benign bone defects in the pediatric population.^134^ A series of similar solutions has been made combining demineralized bone matrix (DBM) particles in similar reverse‐phase medium‐based hydrogels. This includes both Dynagraft® III and AlphaGRAFT® that combines DBM particles with poloxamer gel, meanwhile, AlloFuse® combines DBM particles in a carrier of polyethylene oxide polypropylene oxide block copolymer. Optium® and Grafton® DBM uses glycol as a carrier. Unfortunately, with the exception of Medtronic with their Grafton® product, these manufacturers have chosen to keep their data on file so the products cannot be discussed directly. In general terms, DBM is an attractive biomaterial as the acid‐extraction process allows the retention of growth factors such as bone morphogenic proteins (BMPs), yielding osteoinductive properties, but it is a challenge for manufacturers to sterilize DBMs without inactivating these growth factors.^135^ Due to the risk of immunoreactions and transmission of infections, the use of DBM and other allograft products is regulatorily unfavorable in Europe, and with the new MDR, it is expected to be limited further.^136^

A more recent solution is Anika's Tactoset® solution where calcium phosphate particles and HA are mixed into a hardening, injectable gel solution.^82^ Currently, it has only been published as a technical note with limited information on the composition and therapeutic effect. A similar solution is Globus Medical's Kinex® composed of bioglass and collagen in a HA gel.^83^ However, the manufacturer has chosen to keep their data on file; hence no research is published on this solution.

#### 3.2.3 Orthopedics: Spinal fusion

Spinal fusion is a common surgery requiring bone‐growing implants, with approximately 200,000 lumbar spinal fusions conducted in 2015 in the United States alone.^137^ Spinal fusion is performed to compensate for degenerative disc disease (DDD) where the height of intervertebral disc (IVD) has reduced leading to the compression of the spinal cord, which translates to back pain. Degenerative disc, the first step toward DDD, affects more than 90% of people above 50 years.^138^ When the degeneration progresses, spinal fusion is an attractive surgery for pain mitigation and preventing damage to the spinal cord. The surgery typically consists of a cage being inserted to mechanically regain the spacing between the vertebras, then bone grafts are used to stimulate bone growth to fuse together the adjacent vertebras. Conventionally an open surgery is used, but there is now a trend to use minimally invasive procedures (MIP) such as key‐hole surgery.^139^ MIP can be incompatible with conventional bone grafts due to large size or high viscosity; hence this trend favors injectable solutions such as hydrogels. Moreover, MIP spinal fusion requires less bone to be removed for access to the IVD, which means less autologous bone available as graft material, increasing the demand for alternative grafting materials. Between 9% and 39% of lumbar spinal fusions fail,^140^ indicating a need for more potent bone regrowth solutions. Spinal fusion requires heterotopic ossification, meaning bone tissue growth in soft‐tissue locations where bone is usually not present. This makes it a challenging task, and a graft only exhibiting osteoconductive properties is suboptimal. Ideally, for treatment of large defects and for heterotopic ossification the graft should be osteoinductive, a phenomenon induced when the material creates a local homeostatic imbalance by binding to calcium and/or phosphate ions, causing depletion of these ions.^141^, ^142^ Hence, an osteoinductive material is likely to quickly induce a stable fusion than a graft that is just osteoconductive. This has motivated many to introduce BMP in their graft products, for example, Medtronic uses rhBMP‐2 in their Infuse® (US)/Induct® (EU) bone graft. However, according to the European regulation, the BMP makes it considered a medicinal product. Furthermore, the use of rhBMP‐2 in this product has been linked to several adverse events where the high doses of the growth factor, mainly when used for “off‐label” cervical spinal surgeries, causes an inflammatory effect yielding high complication rates.^143^ A similar BMP‐7 based product named OP‐1® from Stryker has failed to obtain FDA approval for similar spinal applications. However, effective hydrogel therapies are emerging that do not depend on BMP‐based growth factors to obtain their therapeutic effect. The before mentioned Baxter Actifuse® Flow has successfully been used for spinal fusion procedure.^144^ When used in a comparative clinical study to the Medtronic Infuse® graft, they were able to demonstrate similar fusion rates (Actifuse® 9/9, Infuse® 8/9 cases) and both products yielded similar alleviation of pain and improved quality of life.^145^ Also, DBM solutions have been approved clinically for spinal application. The Grafton® DBM was tested in a clinical trial with a total of 120 patients undergoing posterolateral spinal fusion, of which 81 (70%) completed the 24‐month radiographic study.^80^ Grafton® was used on one side of the spine and autograft on the other, and in 42 (52%) of the Grafton® cases, successful fusion was obtained versus 44 (54%) for the autograft side. The authors concluded that the Grafton® DBM gel can be used to extend autograft material during spinal fusion.

### 3.3 Nucleus pulposus

**FIGURE 4:**
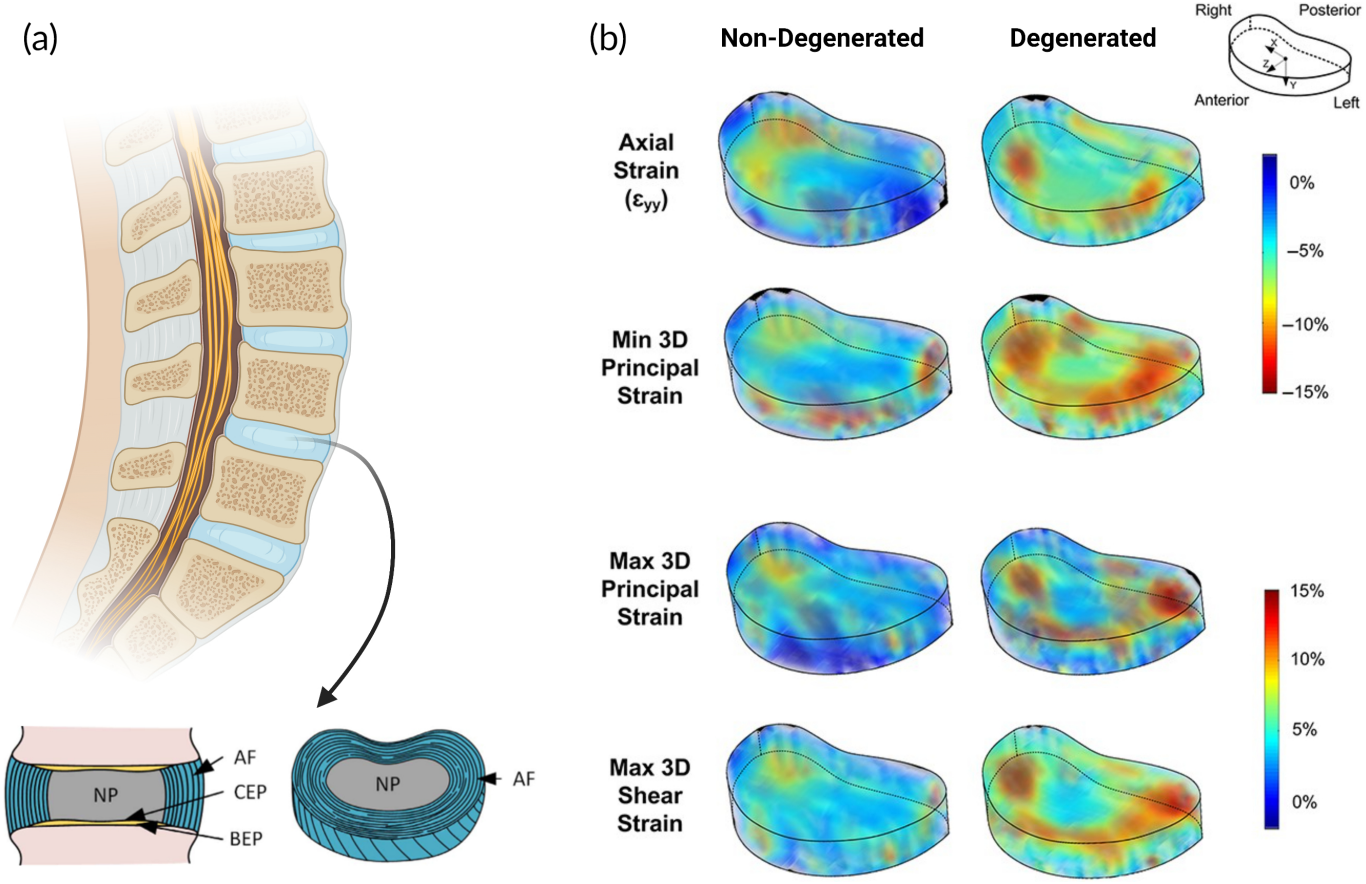
(a) Illustration of the intervertebral disc. (b) Strain and stress levels in non‐degenerated and degenerated intervertebral discs demonstrating how the degenerated disc is prone to higher stress levels, particularly around the AF region. Reproduced and adapted from 137, 138. Reprinted with permission from copyright CC BY 4.0. AF, annulus fibrosus; BEP, bony endplate; CEP, cartilaginous endplate; NP, nucleus pulposus

Spinal fusion tends to be conducted due to DDD, where the IVD has degraded and lost its height or fractured. The IVD is to find between all the vertebra of the spine. It has three main components; the hydrogel‐like NP in the core, surrounded by the annulus fibrosus (AF), and cartilaginous end plates (CEP) at the top and bottom (Figure 4a).^146^

The NP consists of approximately 50% (dry weight) proteoglycan proteins that play a vital role in binding water in the NP and shock absorbance.^147^ During disc degradation, the concentration of proteoglycans decreases, causing a drop in stiffness.^146^ This increases the risk of AF bulging, increases the compressive strain on the AF (Figure 4b), and increases the chances of peripheral failure of the end plates.^148^ Therefore, a potential treatment of DDD would be to repair the NP.

A solution that has been approved for the European market is GelStix®. GelStix® uses a dehydrated polyacrylonitrile that is injected into the NP through a 22‐G needle in the form of a filament, where it gets hydrated from the surrounding body liquids and expands 10‐fold.^84^ In a 12‐month follow‐up with 29 patients, 86.2% rated the procedure as very good or good, and pain relief was observed already after 1 month.^84^ However, there have been reported complications associated with this procedure. Durdag and colleagues reoperated a woman with a GelStix® implanted as she was admitted with severe radicular pain.^149^ The pain was linked to a fragment of implant that had penetrated through an annual tear and caused compression to the spinal root. The authors speculate that the implant may have been initially wrongly placed in the AF, highlighting the importance of the correct placement of the implant.

HA with a similar composition to the solutions used for VS has been used for treatment of the NP. In a 24‐week follow‐up period, Mazza and co‐workers observed relief from chronic lower back pain due to DDD compared to the baseline.^85^ They had two patients drop out due to adverse events, but this is not believed to be related to the treatment. However, the clinical efficacy is proven only over a short time period. Considering the surgical risk related to bypassing vital organs during injection, this therapy can come short when evaluating it using a cost–benefit analysis. Hence a longer‐lasting therapy should be investigated.

Two other solutions have been tried clinically, but seem to have been discontinued. The NuCore® gel for NP replacement consists of elastin and silk co‐polymers that are crosslinked in situ.^89^ A 2‐year follow‐up pilot clinical study with 14 patients demonstrated a significant reduction in back and leg pain, regained disc height, and no side effects.^89^ There have not been any clinical publications on this product since 2009, and it seems to have been discontinued by the supplier. CryoLife started clinical trials on their product BioDisc but have not published the outcome of the trial. In a conference abstract containing interim results, they reported at the 6‐month follow‐up a decrease in mean Oswestry Disability index from 49.2 to 11 and a decrease in numerical pain score from 5.86 to 1.62,^88^ which could seem promising. However, they also reported that 2 of the 10 patients enrolled experienced recurrent herniation requiring surgery. After this abstract from 2008, there has been no publication, and the product seems to have been discontinued.

Since neither CryoLife nor Spine Wave has disclosed why their products were discontinued, it is not feasible to conclude why they failed to perform in the clinic.

## 4 REGULATORY CLASSIFICATION AND CONSEQUENCES

From a regulatory perspective, the first step of translating a medical device is to assign it to the appropriate risk classification group, namely risk class. In Europe with the new MDR, this is reasonably straightforward with injectable hydrogels. Because they are implanted, hence in contact with human tissue over a prolonged period and have a biological effect, it becomes a class III device (highest risk level). This means a premarket clinical investigation is mandatory. This can be mitigated if equivalence to a predicate device can be demonstrated. Nevertheless, appropriate equivalence is practically infeasible unless the manufacturer of the new device either (a) also manufactures the predicate device or (b) has a contractual agreement with the manufacturer of the predicate to access all technical information. In the United States, the risk classification differs from Europe as it depends on product device groups. VS products or dental biologics (e.g., Emdogain®) are class III (highest risk), meanwhile more conventional bone graft materials without human growth factors such as Anika's Tactoset® or the DBM solutions are class II. For class II and some class III product groups the 510(k)‐pathway can be used if it demonstrates substantial equivalence with existing approved devices, demonstrating that the device is safe and efficient, which is significantly cheaper than introducing a new device. In the case of class III, the 510(k) allows the manufacturer to partially bypass the premarket approval application, meaning they do not need to run a clinical investigation, but this is not applicable for the VS products or dental biological materials discussed here. When the 510(k) is not applicable for the class III devices, the product needs to be evaluated on a case‐by‐case basis by the authorities (US‐FDA).

In the review, we have focused on discussing hydrogels as medical devices. However, they can also be classified as medicinal products if their main mechanism of action is through pharmacological, metabolic, or immunological means^6^; this would lead them to the so‐called “drug approval process.” A couple of hydrogels that are used for the above‐described musculoskeletal treatments are classified by the European Medical Agency and the FDA as medicinal products (biologics/drugs) instead of medical devices as they have the characteristics of combinatory products, Advanced Therapy Medicinal Products (ATMP). A summary of these can be found in Table 2.

**TABLE 2:** List of hydrogel solutions for musculoskeletal therapies regulated as medicinal products

Indication and treatment mode	Product (producer)	Composition	Delivery method	Therapeutic claim	FDA approvals
Osteoarthritis					
Cartilage regeneration	NovoCart Inject^150^, ^151^ (Tetec AG)	Maleimide functionalized human albumin and HA crosslinked with bisthio‐PEG, and autologous chondrocytes	Arthroscopic injectable autologous chondrocyte transplant	Needle injection through two‐chamber solution allowing in situ polymerization	FDA phase III trials
DDD					
Nucleus pulposus replacement	NovoCart Disc^152^, ^153^ (Tetec AG)	As above	As above	As above	FDA phase II trials

Abbreviations: DDD, degenerative disc disease; FDA, Food and Drug Administration; HA, hyaluronic acid; PEG, polyethylene glycol.

Over the last couple of decades, there has been a drastic change in the design rationale of orthopedic biomaterials. From passive structures designed for minimal interaction with the surrounding tissue, for example, titanium‐based hip implants, the current generation of biomaterials is designed to actively interact with the surrounding tissue, such as scaffolds for tissue regeneration that stimulates tissue growth. This means that the product's mechanism of action starts approaching that of medicinal products, which will change the applicable regulation framework.^154^ Hence engineers need to carefully consider regulatory classification when designing hydrogels. If a hydrogel solution is classified as a medicinal product, it increases the documentation and overall market entry requirement and requires larger and more costly clinical trials. Compared to medical devices, the therapy will take significantly longer time for clinical translation, the R&D investment costs will increase drastically, and the product will eventually be sold at a higher price to the healthcare providers. Moreover, there will be longer product cycles, which means less innovation. In the United States, it takes on average 12 years from pre‐clinical trials to market approval for drugs while it only takes 3–7 years for medical devices, and the development costs will increase from the range of tens of millions of dollars for medical devices up to the excess of $1 billion for pure drugs.^155^, ^156^

Products where a medical device (i.e., the gel) carries a therapeutic agent such as growth factor or expanded cells no longer gets its primary mode of action through physical means, and the classification changes to medicinal products. For example, Tetac AG (Germany) has developed two such products for cartilage treatment (NovoCart Inject®) and IVD regeneration (NovoCart Disc®). The NovoCart® gel functions as an autologous chondrocyte carrier and is used in a 2‐step surgical procedure, hence is regulated after the complex ATMP framework. In the first step, the chondrocytes are harvested and expanded in GMP facilities. In the second step, the cells are added to a liquid consisting of human albumin and HA. During the injection procedure, the cell/polymer mixture is mixed with a bisthio‐PEG crosslinker which causes the crosslinking of the gel in situ through a Michael‐type addition reaction between the thiol groups of the PEG and the maleimide groups of the functionalized human albumin.^157^ In a short‐term follow‐up (12 months) for cartilage regeneration, they could observe a reduction in pain, an increase in activity and quality of life among the patients.^150^ In a smaller 24‐months study, they demonstrated clinically favorable outcome in terms of reduced pain and a MOCART 2.0 score of 70 ± 13.6, suggesting cartilage regrowth with morphological integrity.^151^ The MOCART 2.0 scoring system uses MRI to quantify the quality of cartilage repair tissue by giving it a score between 0 (worst) to 100 (best).^158^ The Novocart® inject solution has also been tried clinically for NP regeneration. So far, the phase I part of the joint I/II trials have not raised any concerns about the safety of the product.^152^

## 5 FROM LAB TO CLINIC AND EMERGENCE OF POST‐MARKET SURVEILLANCE

**FIGURE 5:**
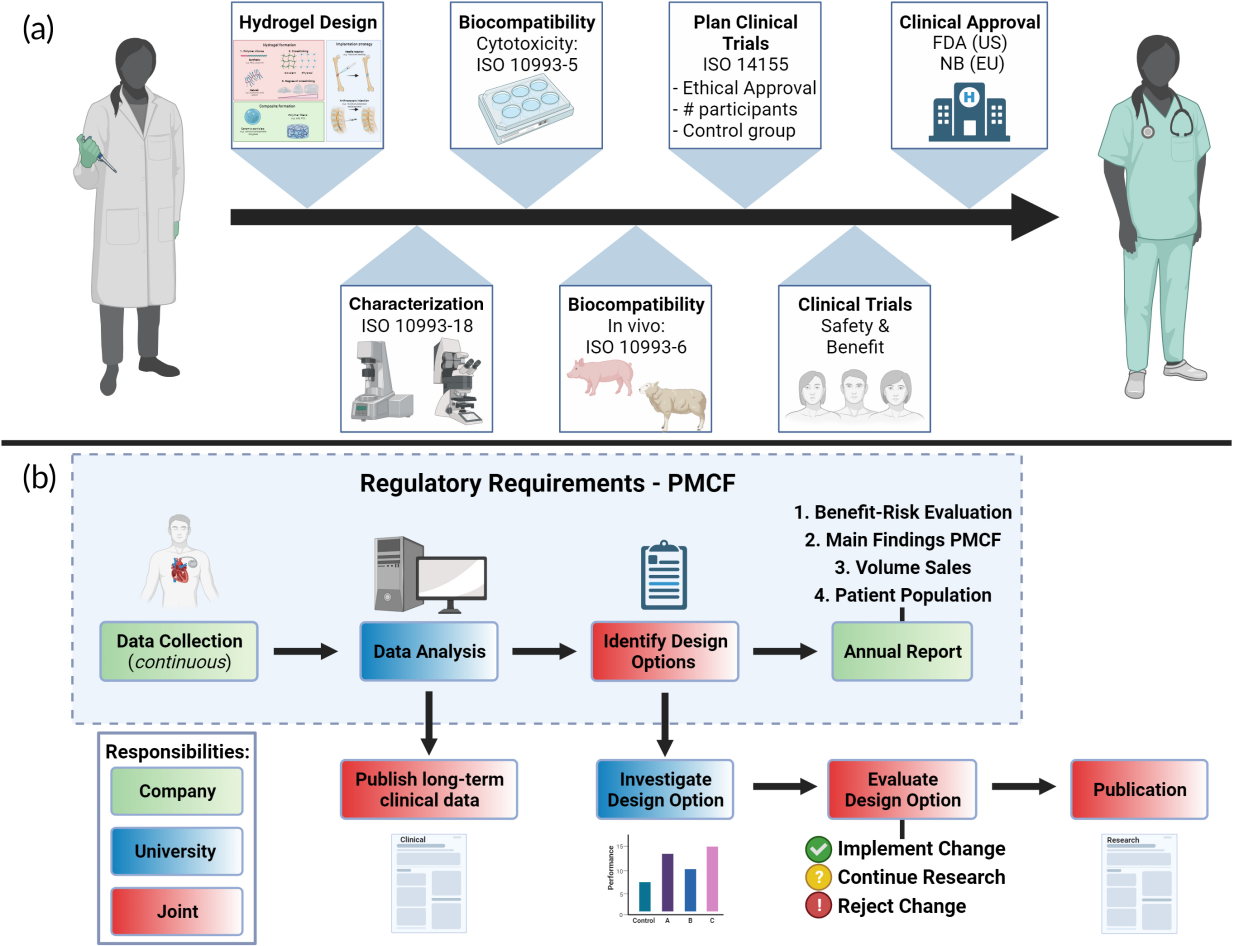
(a) Schematic illustrating the main stages involved in the clinical translation of injectable hydrogels. (b) Suggested framework for industrial‐academic collaboration on post‐market clinical follow‐up (PMCF) in accordance with the requirement of the EU MDR 2017/745 regulations for Class III (high‐risk) medical devices such as hydrogels. The responsibility division is not resolute, with the expectation of the data collection and the annual report, and should be delegated on a case‐to‐case basis. FDA, US Food and Drug Administration; ISO, International Organization for Standardization; NB, notifying body

Translating hydrogels as medical devices is a time‐consuming process, and care should be taken to have a clear plan from design to pre‐clinical and clinical investigation. The steps from hydrogel development to clinical approval and post‐market surveillance have been illustrated in Figure 5. First, the hydrogel needs to be developed; the details of this process have been described above. A thorough material characterization is mandatory for scientific and clinical perspectives and is also useful when explaining the mechanism of action to the notifying body or the US‐FDA. Thereafter, it is mandatory to demonstrate biocompatibility according to the applicable ISO 10993 standards, where the manufacturer must justify which are applicable and which are not. A natural sequence for hydrogels for musculoskeletal application (implantable with long‐term tissue contact) is first characterizing the material's chemical properties according to ISO 10993‐18:2020, then in vitro cytotoxicity according to ISO 10993‐5:2009, and finally pre‐clinical trials according to the ISO 10993‐6:2016 where both the local and systematic response should be evaluated in a reliable animal model. If these are followed diligently and the animal model is well designed, it should cover most of the documentation requirements of the regulatory body, and most of the other ISO 10993 standards can be considered non‐applicable. However, a justification for this must be given in the device's risk management file.

Before the pre‐clinical trials, it is important to have a clear idea of the clinical claims that shall be demonstrated in the animal model stage. Indeed, in pre‐clinical trials, selecting an animal and implantation site that represents the clinical pathophysiology and loading is essential. This has been discussed in detail for injectable bone substitutes by Bongio and colleagues.^159^ If the pre‐clinical trials are successful, it is necessary to go through clinical trials. Since hydrogels tend to be short‐term (>60 min contact with tissue) or long‐term implants (>30 days contact with tissue) with a biological effect, they will be classified as high‐risk (class III) medical devices according to the EU MDR^160^ and require thorough documentation on safety and efficacy. The new EU MDR requires a more comprehensive clinical evaluation than the former regulation, focusing on both direct clinical investigation and literature/market analysis. The specific requirements have been discussed from a notifying body's perspective by Holborow.^161^ It is worth noting that the new regulations require the clinical investigation to have a representative patient group to the EU population, the participation number must be demonstrated statistically to be large enough to be appropriate for demonstrating safety and performance, and the length and follow‐up intervals must give a good picture of the lifetime of the device.^162^ This means that it is technically enough to conduct one clinical trial to get CE approval, but it must be large enough to be exhaustively representative. It is also a requirement that the clinical trials must strictly follow Good Clinical Practise (GCP) guidelines and ISO 14155:2020; hence they must be approved by an ethical committee set up according to national law in the EU member state where the clinical trials are conducted.^162^

The clinical trials for medical devices differ from medicinal products, where there are distinct phases in the clinical trials. The typical set of clinical trials for drugs consists of phase I where safety is demonstrated on a small number of healthy participants, phase II where efficacy is demonstrated on a moderate number of participants, and phase III where efficacy is demonstrated on a larger number of participants. The phase III trial, which ideally is double‐blind and randomized, can involve up to thousands of participants lasting months or years.^163^ This might not be feasible or ethical for medical devices. For example, although saline solution as a control for VS is standard procedure, a sham control for an orthopedic bone graft could do significant damage to the patient and thereby be unethical. For these risk cases, using the current treatment alternative as a positive control could be a good alternative, such as autografts as a control for bone graft substitutes. This allows the manufacturers to benchmark their technology, and it is easier to demonstrate its clinical claims and the value provided to patients and healthcare providers. Since clinical trials directly affect the patient's health, patient safety and ethical standards should be central in clinical trials to reassure a high‐quality standard. The Declaration of Helsinki is an excellent guideline for meeting the ethical standards, together with GCP and ISO 14155:2020.

Notably, the new EU MDR requires a post‐market surveillance register for medical devices (EUDAMED). This is inspired by the successful implementation of orthopedic device registries and the quality of the data these have provided.^4^ With this registry, the regulation requires continuous data gathering and analysis. More specifically the MDR article 83 states^162^: “*The post‐market surveillance system shall be suited to actively and systematically gathering, recording and analysing relevant data on the quality, performance and safety of a device throughout its entire lifetime, and to drawing the necessary conclusions and to determining, implementing and monitoring any preventive and corrective actions*.” The medical device industry is characterized by a lot of small, niche suppliers. In Europe, out of 33,000 medical technology companies, 95% are small or medium enterprises (<250 employees), and a majority are small or micro‐sized companies (<50 employees).^164^ The limited manpower makes it challenging for these companies to designate and dedicate personnel for the post‐market clinical follow‐up. This provides a golden opportunity for academic researchers to collaborate with these companies to analyze the clinical data, and academics can use their understanding of fundamental biological and clinical mechanisms to explain the collected observations, for example, evaluating porcine versus bovine gelatin in the bone graft SmartBone.^165^ If the data are published, it will indeed help the wider research community. Meanwhile, the companies will benefit from this as they can leverage experienced personnel to analyze and explain complex data.

## 6 CONCLUDING REMARK AND FUTURE DIRECTION

There is a tremendous discrepancy between the intensity of academic research on hydrogels and the number of products that have been clinically translated for the treatment of musculoskeletal defects. When developing hydrogels, it is crucial to consider the clinical potential of the material, and here pre‐clinical and clinical trials are key in predicting whether a material candidate will make it past the evaluation of the regulatory body and succeed clinically. On top of that, practical factors such as the cost of the product, scalability, and ease of use in the clinic should be considered at an early point, together with quality assurance and regulatory affairs matters. As demonstrated in this review, the clinically available materials tend to have extensive clinical documentation, but the understanding and documentation of the hydrogel composition tend to be limited. Concurrently, materials that are intensely investigated in academia and have been thoroughly characterized physiochemically and in vitro are not the ones that have made it to the clinic. When searching “GelMA” on PubMed, it yields 701 articles from the last 11 years. Of these, none are clinical trials. The fact that GelMA has not made it to the clinic is likely a consequence that regulatory bodies are primarily concerned about the material's clinical history. Hence, materials that have made it to the clinic before increased documentation requirements are favorable to use in new implants. Meanwhile, new biomaterials are now expensive and scientifically challenging to translate. It can also indicate that academic research environments need to invest more resources to mature the technology through in vitro, pre‐clinical and clinical trials. Particularly, a comprehensive characterization of physiochemical properties, in vitro testing, and use of advanced characterization in animal trials will be helpful for industry, both because it helps them understand the potential of the biomaterial and because it can assist in explaining a device's mechanism of action. A complete understanding of a device's mechanism of action is essential for approval under the new MDR. To increase the likelihood of industrial adoption academics should also demonstrate that any new therapeutic agents can withstand appropriate manufacturing, for instance how an osteoinductive peptide can withstand manufacturing processes with DCM and other solvents,^166^, ^167^ and sterilization processes (e.g., autoclaving, gamma/beta‐irradiation [typically 25 kGy], ethylene oxide) without compromising its efficacy. Additionally, verify that the clinical effect and sterility can be maintained with storage over an extended time period in accordance with ISO 11737‐2:2020.

In vitro testing is very important for understanding isolated mechanisms. However, in our experience^167^, ^168^, ^169^ there are major differences in response to biomaterials during in vitro tests, where single cell types are used, and in vivo, where there is an assortment of cell types interacting.^33^, ^170^ Although there is progress in technology such as organ‐on‐chip^171^, ^172^ or co‐cultures,^173^ they are yet not capable of mimicking the complexity of tissue response to biomaterials. Simultaneously, animal trials should be kept to a minimum for ethical and economic reasons. To obtain adequate documentation and keep animal trials to a minimum, care should be taken in acquiring high quality in vivo data. The ISO 10993‐6 (*Test for local effect after implantation*) requires only local microscopic assessment using histology. Using only this method gives an incomplete picture as conventional histology does not give spatial information or confirm certain biomarkers.^174^ Hence, utilizing additional methods such as cone beam computed tomography,^167^ microCT (μCT),^167^ immunohistochemistry,^167^, ^175^, ^176^, ^177^ small‐angle X‐ray scattering ,^167^, ^178^ X‐Ray diffraction analysis ,^167^, ^178^ and more newly developed techniques such as fluorescent labeling of abundant reactive entities,^167^ optical photothermal infrared microscopy,^167^ and nanoscale atomic force microscopy‐infrared^167^ can give a comprehensive understanding of the material's mechanism of action. Furthermore, there has been an increased focus on the use of intravital microscopy such as fluorescence lifetime imaging microscopy and Raman spectroscopy as their subcellular resolution (approx. 500 nm) allows for studying in detail in vivo host response to implants and for monitoring of implant biology over time in small animal models.^30^ If academics bring their material candidates all the way through animal trials and conduct thorough in vivo characterization, it will assist industrial R&D engineers in making an educated choice of biomaterials in their medical device design. Realizing funding limits related to translational research will require the industry to support the financing of these research activities in active collaborations.

## CONFLICT OF INTEREST

M.M.S. is co‐inventor on patent application 1911235.8 submitted by Imperial College London, related to ultrasound‐triggered liposome payload release.

## AUTHOR CONTRIBUTIONS

**Øystein Øvrebø:** Conceptualization (equal); funding acquisition (equal); visualization (lead); writing – original draft (lead). **Giuseppe Perale:** Conceptualization (equal); supervision (supporting); writing – original draft (supporting). **Jonathan P. Wojciechowski:** Visualization (supporting); writing – review and editing (equal). **Cécile Echalier:** Visualization (supporting); writing – review and editing (equal). **Jonathan R. T. Jeffers:** Supervision (supporting); writing – review and editing (equal). **Molly M. Stevens:** Funding acquisition (equal); supervision (supporting); writing – review and editing (equal). **Håvard J. Haugen:** Conceptualization (equal); funding acquisition (equal); project administration (equal); supervision (equal); writing – original draft (equal). **Filippo Rossi:** Conceptualization (equal); project administration (equal); supervision (equal); writing – original draft (equal).

### PEER REVIEW

The peer review history for this article is available at https://publons.com/publon/10.1002/btm2.10295.

## Data Availability

The PubMed search results on "GelMA" are available upon request from the corresponding author. Otherwise, no data have been used without a reference.
